# Comparison of the femoral condylar ellipse line and the surgical epicondylar axis: 3D measurement by MRI scans in healthy Chinese people

**DOI:** 10.1111/os.13770

**Published:** 2023-06-14

**Authors:** Guanpeng Zhang, Mingyang Liu, Shenghu Fan, Zhaoliang Liu, Xinlin Nie, Xin Qi, Chen Yang

**Affiliations:** ^1^ Department of Orthopaedic Surgery Center The First Hospital of Jilin University Changchun China; ^2^ Department of Orthopaedic Surgery 3nd Xuchang Central Hospital Xuchang China; ^3^ Henan Province Intelligent Orthopedic Technology Innovation and Transformation International Joint Laboratory, Henan Key Laboratory for Intelligent Precision Orthopedics, Department of Surgery of Spine and Spinal Cord Henan Provincial People's Hospital, People's Hospital of Zhengzhou University, People's Hospital of Henan University Zhengzhou China

**Keywords:** Condylar ellipse line, Distal condylar line, Epicondylar axis, Femoral condyle, MRI scan, Posterior condylar line

## Abstract

**Objective:**

The sagittal shapes of the femoral condyles were thought to consist of circles. However, the line connecting the centers of circles was not consistent with the surgical epicondylar axis (SEA) which was commonly used in surgery. Recently, ellipses have been proposed as an alternative method to represent the sagittal femoral condylar shape. Does the condylar ellipse line (CEL) coincide with the SEA in 3D MRI reconstruction analysis?

**Methods:**

From May to August 2021, a total of 80 healthy subjects were scanned by MRI on the right knee in this retrospective study. The ellipses on the most distal slices of the medial and lateral condyles were determined. A line connecting the centers of the medial and lateral ellipses was the CEL. A line connecting the deepest point of the medial sulcus and the most prominent point of the lateral epicondyle was the SEA. Angular measurement of the SEA and the CEL relative to the posterior condylar line (PCL) and the distal condylar line (DCL) was performed on an axial and coronal view of the 3D model, respectively. Measurements were compared between males and females by using the independent‐samples t‐test. Pearson correlation was used to analyze the relationship between SEA‐PCL and CEL‐PCL, SEA‐DCL, and CEL‐DCL.

**Results:**

On the axial view, the mean SEA‐CEL was 0.35° ± 0.96°. SEA‐PCL (2.91° ± 1.40°) had a high correlation with CEL‐PCL (3.27° ± 1.11°) (*r* = 0.731, *p* < 0.001). On the coronal view, the mean coronal SEA‐CEL was 1.35° ± 1.13°. SEA‐DCL (1.35° ± 1.13°) had a low correlation with CEL‐DCL (0.18° ± 0.84°) (*r* = 0.319, *p* = 0.007). On the sagittal view, the outlet points of the CEL on the medial and lateral epicondyles were anatomically located in the anteroinferior direction to the SEA.

**Conclusions:**

CEL traversed the medial and lateral epicondyles, which has a mean deviation of 0.35° with SEA on axial view and a mean deviation of 0.18° with DCL on coronal view. This study suggested that the ellipse approach is an improved scheme for representing the femoral condylar shape.

## Graphical abstract

The angles between the surgical epicondylar axis (SEA), the condylar ellipses line (CEL), the posterior condylar line (PCL), and the distal condylar line (DCL) were measured separately on the axial and coronal view of the 3D distal femoral model.

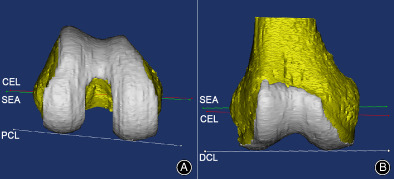

## Introduction

The sagittal geometry of the femoral condyles is closely linked with the kinematics of the tibiofemoral joint. It has been proposed that knee joint flexion occurs around a fixed axis in the femoral condyles.^1^, ^2^, ^3^, ^4^, ^5^ This concept implies that the sagittal geometry of curvature of the femoral condyles is centered on this fixed axis. Some studies have suggested that the sagittal shape of the femoral condyles consists of the posterior circles and the inferior circles, and the posterior circles are centered on the epicondylar axis.^6^, ^7^, ^8^, ^9^ Thus, the epicondylar axis, which passes through the femoral attachments of the medial and lateral collateral ligaments on the epicondyles, has been proposed as a logical reference for proper rotational alignment of the femoral component in total knee arthroplasty.^10^, ^11^ Contrary to the previous statements, further research found that the centers of the posterior condylar circles do not coincide with the epicondylar axis, but pass through the origins of the anterior cruciate ligament and the posterior cruciate ligament.^9^, ^12^, ^13^ Therefore, the epicondylar axis does not appear to be an adequate basis for understanding the shape of the distal femur.^12^ These discrepant statements raise a question: if the epicondylar axis has been repeatedly validated by biomechanical experiments and surgical observations,^14^, ^15^ why does it not coincide with the contour of the femoral condyles?

Recently, Wang et al. proposed a novel ellipse‐fitting approach to simplify the characterization of the sagittal femoral condyle geometry.^16^ When the magnetic resonance images (MRI) scan direction was perpendicular to the surgical epicondylar axis (SEA) on the axial plane and tangent to the distal femoral condylar surface on the coronal plane, a well‐defined ellipse was fitted to both the medial and the lateral femoral condylar cartilage contours. It revealed that the curvatures of the posterior circle and the inferior circle of the medial or the lateral condyle could be perfectly unified into a curvature of an ellipse. Moreover, Zhang et al. compared this elliptical method with the circular method and suggested that both were feasible in representing the sagittal shape of the femoral condyle.^17^

Since the ellipses could represent the sagittal femoral condylar shape, the centers of the condylar ellipse line (CEL) may have a special anatomical correlation with the SEA. Two epicondylar axes have been described in the literature: the clinical epicondylar axis (CEA) and the SEA.^18^, ^19^ The CEA connects the medial and lateral prominences of both epicondyles.^18^ The SEA connects the most prominent point of the lateral epicondyles and the deepest point of the sulcus on the medial epicondyles.^19^, ^20^ In this study, we focused on the SEA because it is more reproducible, and is widely used during total knee arthroplasty.^19^, ^20^, ^21^ On a 3D model of the distal femur, angular measurement of the CEL and SEA was performed on the axial view and the coronal view. The horizontal and vertical distances from the outlet point of the CEL to the SEA were measured on the sagittal MRI slices. The purpose of this study was: (i) to determine the orientation of the CEL by using 3D reconstruction femoral condylar models from MRI data and (ii) to analyze the relationship between the CEL and the SEA.

## Materials and Methods

A total of 82 healthy Chinese volunteers were involved in this study, including 42 males and 40 females. Inclusion criteria were subjects without any history of knee diseased, including (i) trauma, (ii) unfavorable knee symptoms, and (iii) congenital diseases. The exclusion criteria were: (i) presence of abnormal valgus/varus knees (hip‐knee‐ankle angles were >−3^0^ and <3^0^); (ii) discoid meniscus; (iii) trochlear dysplasia; and (iv) cruciate ligaments injury as revealed by MRI images. All signed informed consent forms for MRI examination. This study was approved by our Institutional Review Board (No.2021161).

### Image Acquisition and 3D Reconstruction of the Distal Femur

Sagittal MRI scans of the distal femur were performed on each subject's right knee joint at full extension position using an MR scanner (Philips Achieva 3.0 T magnetic resonance imaging system). The scanning direction was perpendicular to the SEA on the axial plane and was tangential to the distal femoral condylar surface on the coronal plane according to Wang's and Zhang's descriptions.^16^, ^17^ MRI was performed with the following parameters: 3D Water sequence; field of view 180 × 180 mm^2^; 512 × 512 matrix; slice thickness: 1.0 mm; TE: 12 ms; TR: 895 ms. MRI data were imported into Mimics (v.19.0, Materialize, Leuven, Belgium) for segmentation. 3D models of the distal femur and the articular cartilage surface were manually reconstructed. Mimics software was also used to measure the distance and angle. The sensitivity of the measurement was 0.01° for angle and 0.01 millimeter (mm) for length.

### Determination of the Femoral Condylar Ellipses and the CEL

**Fig. 1:**
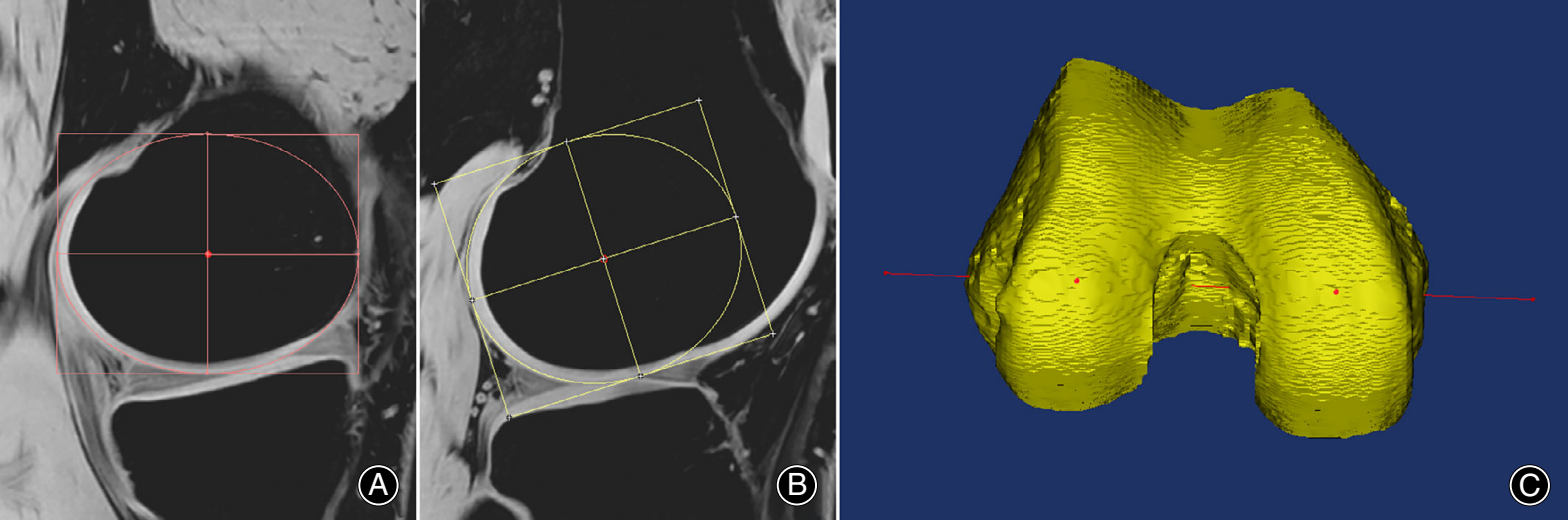
Determination of the femoral condylar ellipses and the CEL. (A) A horizontal ellipse best fitted the articular cartilage surface on the most distal slice of the medial condyle; (B) a rotary ellipse best fitted the articular cartilage surface on the most distal slice of the lateral condyle. The red points represented the centers of the ellipses, respectively; (C) the red line connecting the centers of the medial and lateral ellipses was the CEL.

According to the methods of Wang and Zhang,^16^, ^17^ a horizontal ellipse best fitted the femoral condylar cartilage surface on the middle slice of the medial condyle (usually the most distal slice of each condyle on a coronal view), which had the largest articular surface. On the middle slice of the lateral condyle, a rotational ellipse best fitted the lateral condylar articular surface. In some cases when it was uncertain to determine the lateral middle ellipse, we recommended marking the cartilage surface with a series of “dots”. There was only one ellipse that passed through all the marked “dots” simultaneously. The centers of the condylar ellipses were automatically determined by the Mimics software. The line connecting the centers of the medial and lateral ellipses was defined as the CEL (Fig. 1A–C). The parameters of the major axis and the minor axis of the ellipses were automatically represented by the Mimics software.

### Determination of the SEA

**Fig. 2:**
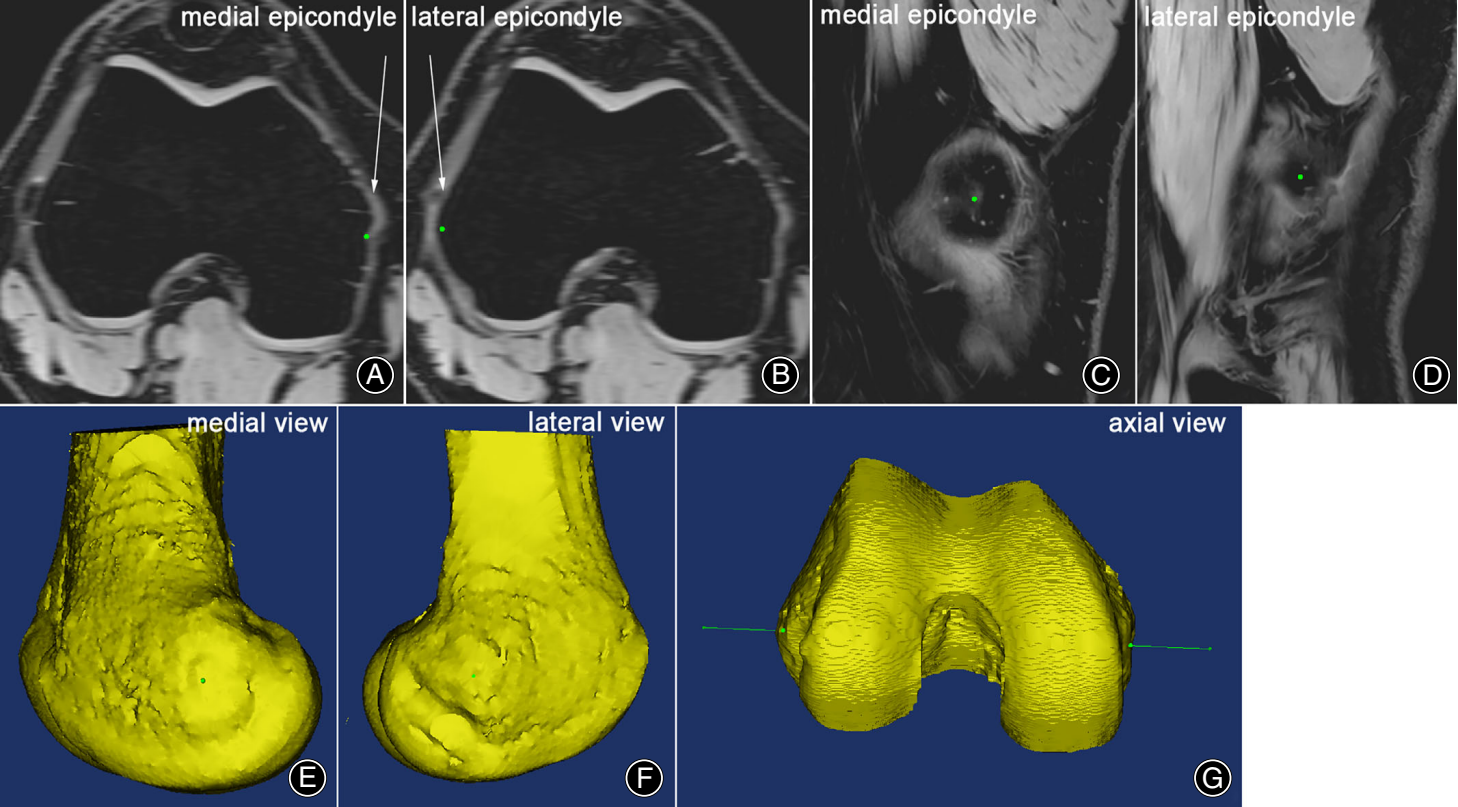
A 3D model and an MRI are used to determine the SEA. (A and B) The lateral epicondyle's most prominent point and the medial epicondyle's deepest sulcus point were both marked (the green points), and both locations were confirmed on the MRI sagittal slices (C and D). Noted that the central sulcus was surrounded by a horseshoe‐shaped bony ridge; (E and F) Both points were verified on the 3D model; (G) The green line linking the two green points was SEA.

According to Akagi's method in determining the SEA, the most prominent point of the lateral epicondyles was marked on the consecutive axial slices.^22^ The accurate position of this point was confirmed by reference to the images on the sagittal slices and the 3D distal femoral model. Similarly, the deepest point of the sulcus on the medial epicondyles was marked and verified. A line connecting the above two points was defined as the SEA (Fig. 2A–G). Three examiners agreed on the determination of the SEA.

### Measurement on the 3D Femoral Model

A line tangential to the cartilage surface of the posterior condyles (posterior condylar line, PCL) and a line tangential to the cartilage surface of the distal condyles (distal condylar line, DCL) were drawn according to previous reports. Angular measurement was performed between the SEA and the CEL on both the axial and coronal views of the 3D femoral model.

**Fig. 3:**
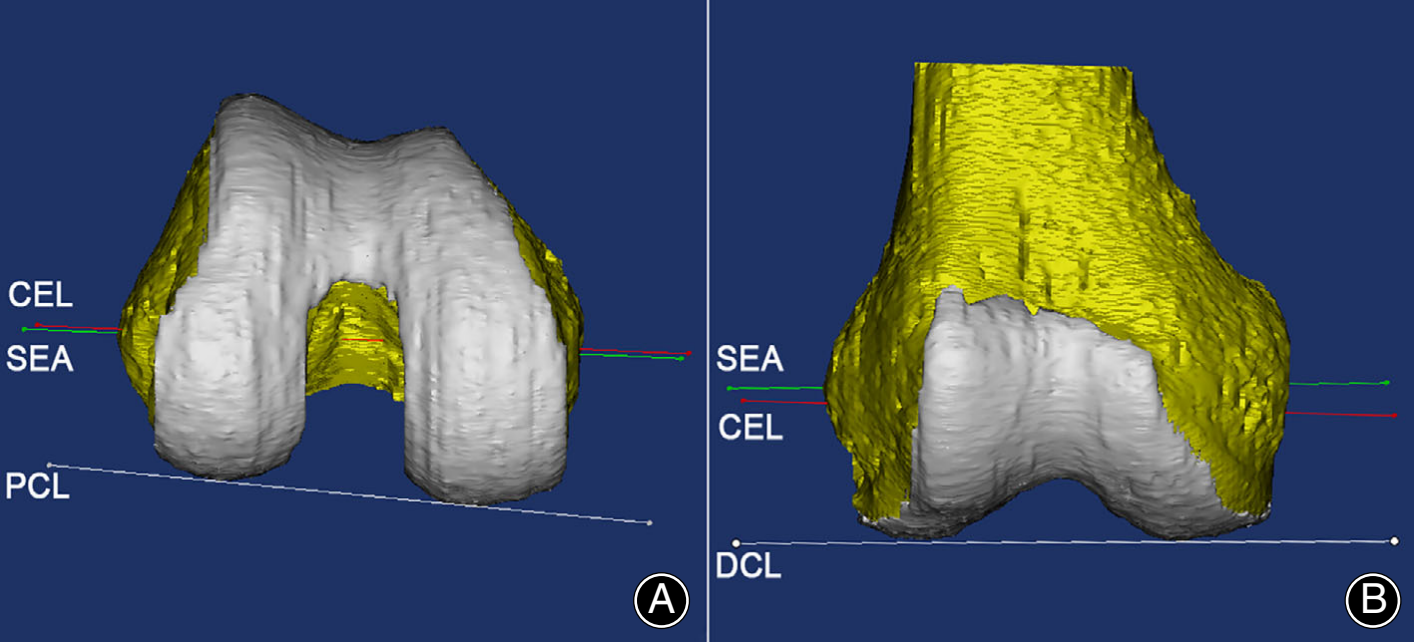
The angular measurement of SEA and CEL on the axial and the coronal view on the 3D distal femoral model. (A) On the axial view, the angles of SEA‐PCL, CEL‐PCL, and SEA‐CEL were measured, respectively; (B) On the coronal view, SEA‐DCL, CEL‐DCL, and SEA‐CEL were measured, respectively. The red line was the CEL, and the green line was the SEA. PCL: the posterior condylar line; DCL, the distal condylar line.

**Fig. 4:**
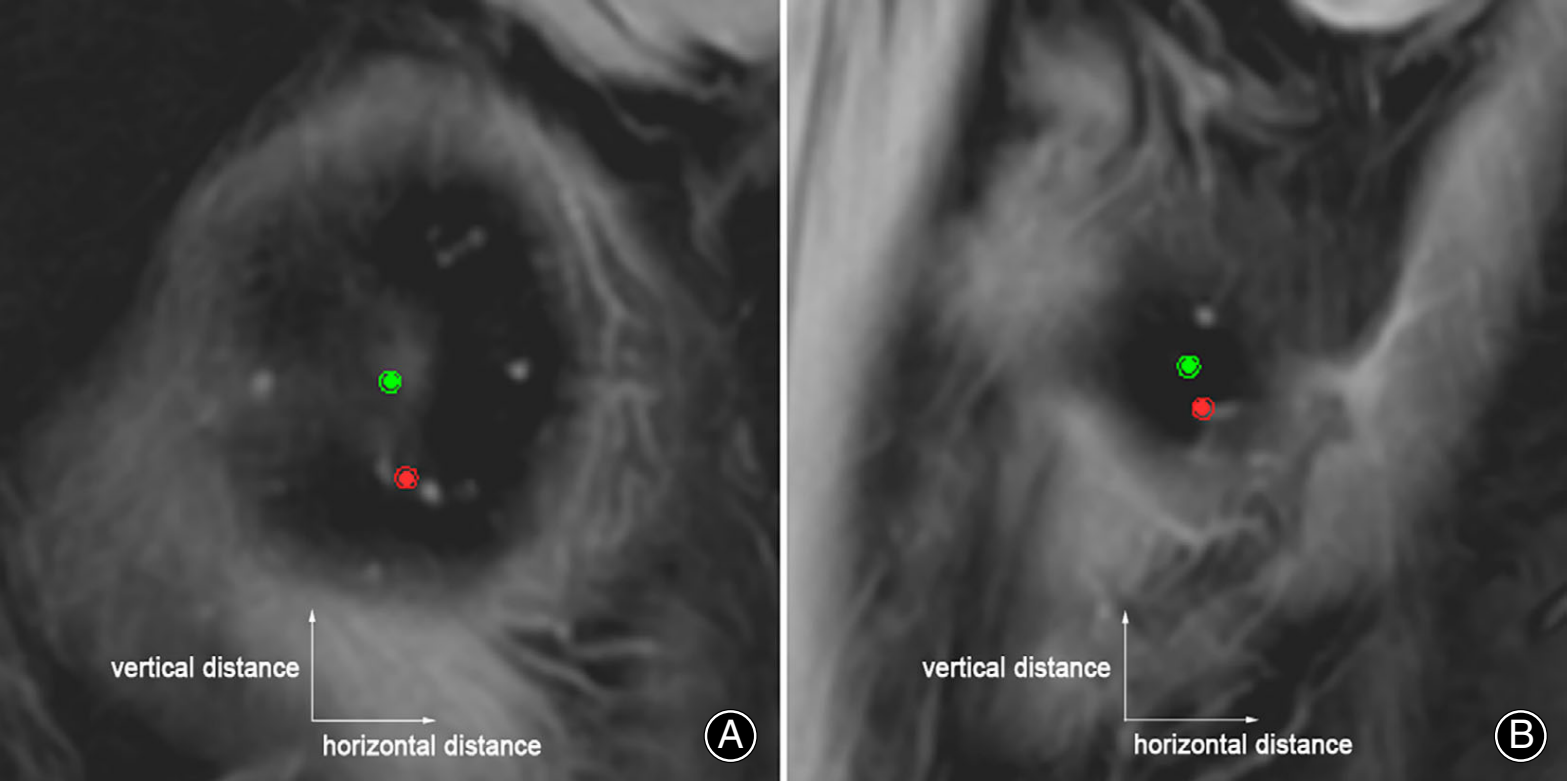
The distance measurement of the outlet points from SEA to CEL on the sagittal slices. (A and B) The horizontal and vertical distances of the outlet points from SEA to CEL on the medial and lateral epicondyles were measured, respectively. Red point: the outlet of CEL; Green point: the outlet of SEA.

On the axial view, the angular measurement was performed on SEA‐PCL, CEL‐PCL, and SEA‐CEL. A positive value was assigned if the SEA or CEL was externally rotated relative to the PCL, or otherwise it was negative. When measuring SEA‐CEL, a positive value was assigned if CEL was in external rotation relative to the SEA. On the coronal view, SEA‐DCL, CEL‐DCL, and SEA‐CEL were measured (Fig. 3A,B). For the DCL, varus was recorded as positive, and valgus as negative. A positive value for the CEL was varus to the SEA when SEA‐CEL was measured on the coronal plane. On MRI sagittal slices, the horizontal and vertical distances of the outlet points from SEA to CEL were measured on the medial and lateral epicondyles, respectively (Fig. 4A,B). To determine the intra‐ and inter‐observer reliabilities of the measurements, one of the authors repeated measurements of the axial SEA‐CEL, and the coronal SEA‐CEL in all subjects at 14‐day intervals. Two of the authors performed blinded measurements of the axial SEA‐CEL, and the coronal SEA‐CEL, respectively.

### Statistical Analysis

The reliabilities of intra‐ and inter‐observer measurements were assessed using intra‐class correlation coefficients (ICCs). Measurement data conforming to normal distribution were expressed as mean ± standard deviation (mean ± SD). Measurements were compared between males and females by using the independent‐samples *t‐*test. Pearson correlation was used to analyze the relationship between SEA‐PCL and CEL‐PCL, SEA‐DCL, and CEL‐DCL. A difference of p < 0.05 was considered statistically significant. A correlation was classified by high (r > 0.7), moderate (0.7 > r > 0.4), or low (r < 0.4) grade. All statistical analyses were performed with SPSS 21.0 software (IBM, Armonk, NY).

## Results

### General information

Two female subjects failed to show identifiable medial sulcus on the MRI. As a result, SEA could not be determined. Finally, 42 males (mean 25.5 ± 1.9 years) and 38 females (mean 26.2 ± 1.4 years) were included in this study. The mean height was 174.8 ± 5.5 cm for males, and 162.1 ± 4.8 cm for females. The mean Body Mass Index (BMI) was 24.66 ± 3.45 kg/m^2^ for males and 20.13 ± 2.57 kg/m^2^ for females. The mean values of the major axis and the minor axis of the medial condylar ellipse were 55.50 ± 4.30 mm and 44.21 ± 3.60 mm, respectively. The mean values of the major axis and the minor axis of the lateral condylar ellipse were 50.60 ± 4.03 mm and 44.22 ± 3.89 mm, respectively.

### Reliabilities

Intraobserver reliabilities for measurements of the axial SEA‐CEL, and the coronal SEA‐CEL were 0.86 and 0.83, respectively. Interobserver reliabilities for measurements of these parameters were 0.78 and 0.76, respectively.

### Imaging Findings and Correlations

**Fig. 5:**
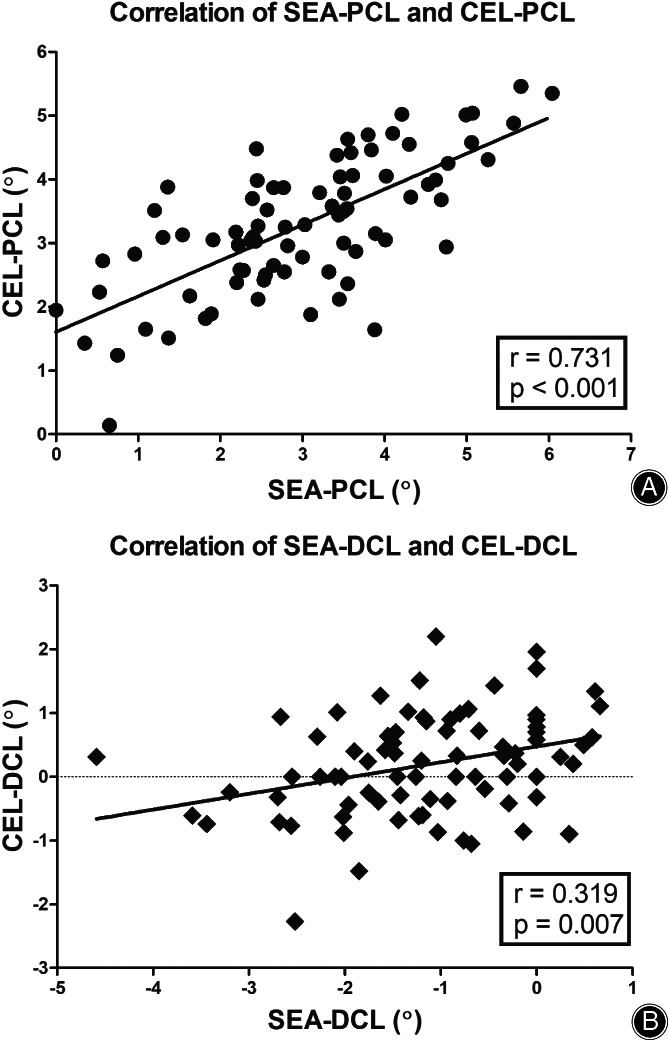
Correlation analysis of the SEA‐PCL and CEL‐PCL, SEA‐DCL, and CEL‐DCL, respectively. (A) SEA‐PCL had a high correlation with CEL‐PCL (*r* = 0.731, *p* < 0.001); (B) SEA‐DCL had a low correlation with CEL‐DCL (*r* = 0.319, *p* = 0.007). CEL: condylar ellipse line; SEA: surgical epicondylar axis; PCL: posterior condylar line; DCL: distal condylar line.

**Fig. 6:**
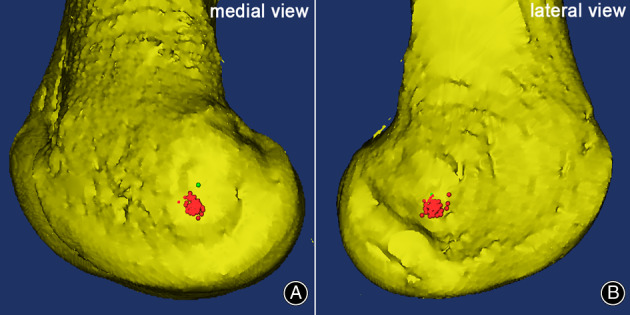
The superimposition of CEL's points that were scaled according to the elliptical parameters on the 3D distal femoral model. (A) On the sagittal view, the outlet points of CEL on the medial epicondyle were anatomically located on the anteroinferior direction to SEA. (B) The outlet points of CEL on the lateral epicondyle were approximately located on the anteroinferior direction to SEA. Red points: the outlets of CEL; Green points: the outlets of SEA.

On the axial view, SEA‐PCL (2.91° ± 1.40°) had a high correlation with CEL‐PCL (3.27° ± 1.11°) (*r* = 0.731, *p* < 0.001) (Table 1, Fig. 5A), and the mean SEA‐CEL was 0.35° ± 0.96° (95% CI: 0.13–0.58) (Table 1). On the coronal view, SEA‐DCL (1.35° ± 1.13°) had a low correlation with CEL‐DCL (0.18° ± 0.84°) (*r* = 0.319, *p* = 0.007) (Table 1, Fig. 5B), and the mean coronal SEA‐CEL was 1.35° ± 1.13° (95% CI: 1.07–1.62) (Table 1). On the sagittal view, the outlet point of the CEL on the medial epicondyle is anatomically positioned in the anteroinferior direction of the SEA (the horizontal distance: 1.01 ± 0.92 mm, the vertical distance: 4.62 ± 1.16 mm) (Table 1). It was close to the anteroinferior “horseshoe ridge” of the medial epicondyle (Fig. 6A). The outlet point of the CEL on the lateral epicondyle is approximately located in the anteroinferior direction to the SEA (the horizontal distance: 0.56 ± 1.17 mm, the vertical distance: 2.80 ± 1.14 mm) (Table 1, Fig. 6B).

**TABLE 1:** Angulation of CEL, SEA, PCL, and DCL on axial and coronal plane (°) (mean ± SD).

	Average	Male	Female	*p* value
Axial CEL‐PCL (°)	3.27 ± 1.11	3.26 ± 1.17	3.29 ± 1.04	0.887
Axial SEA‐PCL (°)	2.92 ± 1.40	3.05 ± 1.46	2.77 ± 1.32	0.410
Axial CEL‐SEA (°)	0.35 ± 0.96	0.21 ± 0.95	0.52 ± 0.95	0.170
Coronal CEL‐DCL (°)	0.18 ± 0.84	0.29 ± 0.77	0.06 ± 0.91	0.251
Coronal SEA‐DCL (°)	−1.17 ± 1.06	−1.06 ± 1.12	−1.28 ± 1.00	0.395
Coronal CEL‐SEA (°)	1.35 ± 1.13	1.35 ± 1.16	1.34 ± 1.10	0.959
Medial horizontal distance (mm)	1.01 ± 0.92	0.95 ± 0.83	1.08 ± 1.03	0.544
Medial vertical distance (mm)	4.62 ± 1.16	4.78 ± 1.13	4.43 ± 1.17	0.207
Lateral horizontal distance (mm)	0.56 ± 1.17	0.68 ± 1.20	0.42 ± 1.13	0.353
Lateral vertical distance (mm)	2.80 ± 1.14	2.81 ± 1.26	2.78 ± 1.02	0.921

*Note*: The sagittal horizontal and vertical distances of the outlet points from SEA to CEL on the medial and lateral epicondyles (mm) (mean ± SD).

Abbreviations: CEL, condylar ellipse line; DCL, distal condylar line; PCL, posterior condylar line; SEA, surgical epicondylar axis.

## Discussion

### Research Findings

The current study revealed valuable findings. First, we determined the orientation of the CEL on 3D MRI and found that the sagittal curvature of the femoral condyles could be best fitted by ellipses. Second, we investigated the relationship between the CEL and the SEA. We found the CEL traversed the medial and lateral epicondyles and had a mean deviation of 0.35° to the SEA on the axial plane and a mean deviation of 1.35° to the SEA on the coronal plane. The CEL was also approximately parallel to the DCL (0.18° ± 0.84°). All of these observations suggested that the ellipse approach is an improved scheme for representing the femoral condylar shape.

### Geometry of the Femoral Condyles

It is widely accepted that the sagittal geometry of the femoral condyle consists of a posterior circle and an inferior circle. During the knee flexion, the arc of the inferior circle contacts the tibial side from 20° to full extension, and the arc of the posterior circle contacts from 20° to 150° of flexion.^23^ Although the posterior circle measurements were consistent, the inferior circle measurements were variable.^24^, ^25^, ^26^ Wang et al. proposed an ellipse approach to characterize the curvature of the femoral condyles that consolidated the curves of the posterior and the inferior circles. They suggested that this approach resolved the variations in the inferior circle measurements and simplified the characterization of the sagittal femoral condyle geometry.^16^ Subsequently, Zhang et al. investigated the relationship between the sagittal contour of the femoral condyle and the posterior tibial slope by using ellipses and circles.^17^ They found that both the medial femoral condylar ellipses and circles strongly correlated with the medial posterior tibial slope, indicating that the ellipse approach was appropriate and logical. In the present study, we confirmed that the ellipse‐fitting scheme is a novel and superior way to describe the sagittal articular surfaces of the femoral condylar curvatures. Our parameters of the ellipses were consistent with those of Wang et al. or Zhang et al., demonstrating that the ellipse method is reproducible and reliable.

### Kinematics of Femoral Condyles

The medial and lateral femoral epicondyles are important anatomical landmarks of the distal femur, serving as the attachment points for the medial and lateral collateral ligaments, respectively. Some in vitro and in vivo studies have shown that the posterior condyles appear circular and the centers pass through the medial and lateral femoral epicondyles, which coincides with the epicondylar axis.^1^, ^9^ Therefore, the epicondylar axis has been proposed as a reliable reference for determining the rotational alignment of the femoral component in total knee arthroplasty.^21^ However, some studies have come to a different conclusion, that the epicondylar axis does not coincide with the contour of the femoral condyles.^12^, ^13^, ^27^ Lustig et al. investigated the existence of a circle centering on the epicondyles and fitting the whole femoral contours. However, such circles could not be identified in any of the specimens, either on the medial or the lateral side.^13^ Eckhoff et al. showed that the posterior femoral condyles closely simulated a cylinder with a single axis. This cylindrical axis passed through the origins of the ACL and PCL on the sagittal MRI but was distinctly different from the epicondylar axis.^12^, ^27^ Therefore, according to these studies, the epicondylar axis does not appear to be an adequate basis for understanding the shape of the distal femur. These contradictory statements have not been clearly explained. The epicondylar axis has been demonstrated by biomechanical experiments as “an optimal flexion axis” and has been validated by surgical observations.^9^, ^14^, ^15^, ^28^, ^29^, ^30^ However, it does not coincide with the centers of the posterior femoral condyles. The posterior condylar centers showed little benefit for total knee arthroplasty operations but were widely used to analyze the kinematics of the knee. It is particularly important to recognize that selection on different reference axes would lead to different kinematic descriptions on the tibiofemoral joint. The characteristics of the kinematics of the femoral condyles are “rotation and rollback”, and the centers of the posterior condyles are “medial pivoting”.^6^, ^23^, ^31^, ^32^, ^33^, ^34^ This distinction has important implications for guiding the design of knee joint prostheses. In this study, the CEL traversed the medial and lateral epicondyles, and approximately coincided with the SEA (average 0.35°) on the axial plane and approximately paralleled the DCL (average 0.18°), which represented the knee joint line on the coronal plane.

### Anatomy of Medial Collateral Ligament Attachment

There is consensus that the medial epicondyle is characteristically composed of a bony ridge surrounding a central sulcus. The bony ridge has been described as a “crescent” or “inverted horseshoe” shape.^13^, ^28^, ^35^ Some studies have reported that the superficial medial collateral ligament is directly attached to the medial epicondyle.^36^, ^37^, ^38^, ^39^ Berger et al. examined 75 embalmed anatomic specimens and showed that the superficial medial collateral ligament attached to the crescent ridge and the deep medial collateral ligament attached to the medial sulcus.^28^ Griffin et al. and Lustig et al. showed that the attachment of the deep medial collateral ligament was located in the medial sulcus, and the superficial medial collateral ligament was located proximal to the deep medial collateral ligament.^13^, ^35^ In contrast, La Prade et al. and Wijdicks et al. found that the attachment of the superficial medial collateral ligament was centered in the medial sulcus which was slightly proximal and posterior to the medial epicondyle. The deep medial collateral ligament has a slightly curved convex attachment that was distal and deep to the femoral attachment of the superficial medial collateral ligament.^40^, ^41^ In a cadaveric study, Liu et al. showed that the attachment of the anterior portion of the superficial medial collateral ligament covered the medial epicondyle and the deep medial collateral ligament was inferior to it.^42^ In the present study, the CEL on the medial epicondyle was located 4.62 mm below the medial sulcus, just at the inferior ridge of the horseshoe. According to the above descriptions,^40^, ^41^, ^42^ the inferior ridge of the horseshoe was most likely the main attachment site for the deep medial collateral ligament, but not for the superior medial collateral ligament. Some studies have shown that the deep medial collateral ligament plays an important role in knee stability, especially in rotation.^43^, ^44^ Our study also indicated that the deep medial collateral ligament might be an important structure on the medial condyle.

### Strengths and Limitations

This study indicated that the ellipse approach is an improved scheme for representing the femoral condylar geometry. CEL has certain guiding significance for the placement of patellofemoral joint prosthesis. And it is also helpful for the preoperative design of unicompartmental knee arthroplasty. There were certain limitations in this study. First, all the subjects were from a Chinese population with anatomical differences from other racial or ethnic groups. Second, only 80 subjects were recruited. In future studies, a larger sample size should be used to determine whether the CEL correlates with varus or valgus. Third, all the healthy subjects were young adults with a mean age of 25.7 years. Older groups may have different elliptical parameters and anatomical relationships between the CEL and the SEA. Lastly, the CEL is better determined by preoperative 3D MRI. Although the use of 3D MRI is not uncommon in knee arthroplasty as the progression of precision and personalization treatment nowadays, it can still incur additional expense for patients.

### Conclusions

The present study supported the theory that the sagittal curvature of the femoral condyles could be best fitted by ellipses. The medial and lateral epicondyles were traversed by the CEL, which was only 0.35° (almost parallel) to the SEA on the axial view and only 0.18° to the DCL on the coronal view. Our study demonstrated that the ellipse approach is an improved scheme for representing the femoral condylar shape.

## Author Contributions

The concept of the manuscript was devised by Chen Yang. Shenghu Fan, Zhaoliang Liu, and Xinlin Nie performed the overall literature searches. Guanpeng Zhang and Mingyang Liu were in charge of writing. Tables and Figure were designed by Guanpeng Zhang. Statistical Analysis was performed by Guanpeng Zhang and Chen Yang. Xin Qi and Chen Yang discussed the content of the article and gave suggestions.

## Conflict of Interest Statement

There is no conflict of interest.

## Funding Information

Jilin Province Science and Technology (Nos. 20200201407JC); The Scientific and technological achievements transformation project of The First Hospital of Jilin University (JDYY2021‐A0004).
